# Efficient classification of complete parameter regions based on semidefinite programming

**DOI:** 10.1186/1471-2105-8-12

**Published:** 2007-01-15

**Authors:** Lars Kuepfer, Uwe Sauer, Pablo A Parrilo

**Affiliations:** 1 Institute of Molecular Systems Biology, ETH Zürich, CH-8093 Zürich, Switzerland; 2 Laboratory for Information and Decision Systems, Massachusetts Institute of Technology, Cambridge MA 02139, USA

## Abstract

**Background:**

Current approaches to parameter estimation are often inappropriate or inconvenient for the modelling of complex biological systems. For systems described by nonlinear equations, the conventional approach is to first numerically integrate the model, and then, in a second *a posteriori *step, check for consistency with experimental constraints. Hence, only single parameter sets can be considered at a time. Consequently, it is impossible to conclude that the "best" solution was identified or that no good solution exists, because parameter spaces typically cannot be explored in a reasonable amount of time.

**Results:**

We introduce a novel approach based on semidefinite programming to directly identify consistent steady state concentrations for systems consisting of mass action kinetics, i.e., polynomial equations and inequality constraints. The duality properties of semidefinite programming allow to rigorously certify infeasibility for whole regions of parameter space, thus enabling the simultaneous multi-dimensional analysis of entire parameter sets.

**Conclusion:**

Our algorithm reduces the computational effort of parameter estimation by several orders of magnitude, as illustrated through conceptual sample problems. Of particular relevance for systems biology, the approach can discriminate between structurally different candidate models by proving inconsistency with the available data.

## Background

Systems biology is a framework integrating diverse disciplines such as molecular biology and genetics with mathematical modelling and simulation, where computational models assume an increasingly important role in recent years. Simulations are an essential element for quantitative understanding because the highly nonlinear behavior of complex biological systems can sometimes be counterintuitive [1,2]. System identification, which comprises both parameter estimation and structural network analysis in biological systems, can be extremely difficult since the governing principles and topological interactions are often not known [3]. The flood of data from novel high-throughput and genome-wide analyses further adds to the problem, and their diversity poses additional challenges for consistency testing and integration. Exact values of kinetic parameters (e.g., association or dissociation constants for protein-protein interaction) are very difficult to determine experimentally, because they are a function of the heterogeneous spatial and developmental cellular conditions.

Most approaches to mathematical modelling of biological systems either avoid parametrization by focusing on static steady-state models of reaction stoichiometry or topology [4,5], approximate behavior and regulation through heuristic-based approaches [6,7], or reduce the solution space by applying enzyme kinetics such as in traditional Michaelis-Menten or *linlog *kinetics, respectively [8,9]. While this enables the detailed modelling of time-dependent processes [10], the underlying quasi steady-state assumption *a priori *neglects the dynamics of enzyme complexes. Thus, in many cases the use of mass-action kinetics in the form of elementary reactions becomes necessary, for instance when some of the enzyme kinetics do not follow Michaelis-Menten [11], a steady-state of the system cannot be assumed [12], transport functions have to be described [13] or the network structure is uncertain [14,15].

The systems of equations that arise in time-dependent models based on chemo-physical kinetics of any kind are usually hard to parameterize, since their components are tightly coupled and there is limited information about the time evolution of the concentrations of all the species. Several approaches for efficient system identification have been developed, ranging from reduction of system dimensionality [16] to decoupling of the differential equations [17]. The actual parametrization algorithm, here referred to as the *wrapping algorithm*, is of particular importance [18] and was for example highlighted in a comparison between gradient-based methods and genetic and evolutionary algorithms [19]. All of these approaches however consider single, isolated points in the parameter space; this can be very time-consuming due to the iterative nature of the parametrization process [19] and furthermore cannot guarantee that the algorithm finds possible solutions.

We present here a conceptually novel approach based on techniques from numerical convex optimization, in particular *semidefinite programming *(*SDP*) [20], that can efficiently partition the parameter space into feasible and infeasible regions. Basically, our approach allows to analyze sets of mass action kinetics, i.e., ordinary differential equations (ODEs) at steady state, under consideration of additional algebraic equality and inequality constraints (e.g., mass balances or formation rates). It is thus possible to simultaneously consider all the available information during the parameter estimation itself, rather than checking consistency *a posteriori *as in most current methods.

Previous applications used SDP either for rational experimental design based on covariance analysis [21] or for the derivation of barrier certificates by construction of surrogate models [22]. In contrast, the algorithm presented here needs no problem reformulation but is based directly on the original parameter estimation problem. By exploiting convexity in the search for feasible steady state concentrations, our methods enable a multi-dimensional perturbation analysis for *sets *of parameters. Our algorithm therefore provides rigorous proofs for the classification of the parameter space into feasible and infeasible regions, thus reducing the number of points needed for consideration by several orders of magnitude.

## Methods

### Steady state analysis

The natural starting point in the analysis of dynamical systems in biology is the determination of a steady state equilibrium of the model, since it represents the reference point for any kind of perturbation introduced to the system. It is therefore of utmost importance that the species' concentrations at an equilibrium point, which is henceforth synonymously referred to as steady state, are realistic and validated against as much data as possible.

In the present study we consider models in the form of mass action kinetics, hence all reaction rates are direct functions of the concentrations *y *and the kinetic constants *k*. At an equilibrium point, the corresponding system of *n *ODEs reduces to a set of polynomial equations

*f*_*i *_(*k, y*) = 0,     *i *= 1, ..., *n*.     (1)

Besides the equations above, the state concentrations also have to satisfy various kinds of constraints based on experimental data, e.g., formation rates or overall mass balances. Since these will in practice inevitably include certain errors, *inequality *constraints also arise naturally. Consider for instance the general mass balance equations, here described by a matrix *M*, which when multiplied with the array of state variables *y *satisfy

*M*·*y *= *b*,     *M *∈ ℝ^*m *× *n*^,     (2)

where *b *∈ ℝ^*m *^is the total amount of each species. Hence, Eqn. (2) represents overall mass balances while Eqn. (1) comprises the mass action kinetics, i.e., the formation and consumption rates of each single species. Since components can exist both unbound or bound in a complex, respectively, there are in general fewer mass balance equations than differential equations (*n *≥ *m*). An experimental error *ε *can be taken into account by relaxing the exact mass balance equations to inequalities where

(1 - *ε*)·*b *≤ *M*·*y *≤ (1 + *ε*)·*b*.     (3)

Thus, the parameter estimation problem can be rewritten as a nonlinear optimization

min⁡yf0(k,y)s.t.fi(k,y)=0gi(k,y)≥0,     (4)
 MathType@MTEF@5@5@+=feaafiart1ev1aaatCvAUfKttLearuWrP9MDH5MBPbIqV92AaeXatLxBI9gBaebbnrfifHhDYfgasaacH8akY=wiFfYdH8Gipec8Eeeu0xXdbba9frFj0=OqFfea0dXdd9vqai=hGuQ8kuc9pgc9s8qqaq=dirpe0xb9q8qiLsFr0=vr0=vr0dc8meaabaqaciaacaGaaeqabaqabeGadaaakeaafaqaaeWacaaabaWaaCbeaeaacyGGTbqBcqGGPbqAcqGGUbGBaSqaaiabdMha5bqabaaakeaacqWGMbGzdaWgaaWcbaGaeGimaadabeaakiabcIcaOiabdUgaRjabcYcaSiabdMha5jabcMcaPaqaaiabbohaZjabc6caUiabbsha0jabc6caUaqaaiabdAgaMnaaBaaaleaacqWGPbqAaeqaaOGaeiikaGIaem4AaSMaeiilaWIaemyEaKNaeiykaKIaeyypa0JaeGimaadabaaabaGaem4zaC2aaSbaaSqaaiabdMgaPbqabaGccqGGOaakcqWGRbWAcqGGSaalcqWG5bqEcqGGPaqkcqGHLjYScqaIWaamcqGGSaalaaGaaCzcaiaaxMaadaqadaqaaiabisda0aGaayjkaiaawMcaaaaa@5918@

that depends on the steady state concentrations *y *and the set of kinetic parameters *k*. Here, *f*_0 _(*k, y*) is an arbitrary objective function, e.g., the overall model error, the equality constraints *f*_*i *_(*k, y*) represent the steady state condition and *g *(*k, y*) is a set of inequality constraints, e.g., the mass balances.

### Semidefinite programming

In the following, we will introduce a reformulation of the generalized parameter estimation problem (4) based on semidefinite programming (SDP). SDP is a specific kind of convex optimization problem [20,23,24], with very appealing numerical properties. An SDP problem corresponds to the optimization of a linear function subject to a matrix inequality. The only prerequisite for the applicability of the SDP is a quadratic (or polynomial) representation of the original set of equalities and inequalities (4), which allows a reformulation/relaxation in terms of symmetric matrices. For clarity of exposition, we focus on the case where the *f*_*i *_are quadratic and the *g*_*i *_are linear, although our methods extend to the fully polynomial case; see [25] for details.

For the SDP relaxation we define new variables *x, X *in terms of the original state variables *y *by:

x=[1y]∈ℝn+1,X=x⋅xT=[1yTyyyT]∈ℝ(n+1)×(n+1).     (5)
 MathType@MTEF@5@5@+=feaafiart1ev1aaatCvAUfKttLearuWrP9MDH5MBPbIqV92AaeXatLxBI9gBaebbnrfifHhDYfgasaacH8akY=wiFfYdH8Gipec8Eeeu0xXdbba9frFj0=OqFfea0dXdd9vqai=hGuQ8kuc9pgc9s8qqaq=dirpe0xb9q8qiLsFr0=vr0=vr0dc8meaabaqaciaacaGaaeqabaqabeGadaaakeaafaqabeqacaaabaGaemiEaGNaeyypa0ZaamWaaeaafaqabeGabaaabaGaeGymaedabaGaemyEaKhaaaGaay5waiaaw2faaiabgIGioprr1ngBPrwtHrhAYaqeguuDJXwAKbstHrhAGq1DVbaceaGae8xhHi1aaWbaaSqabeaacqWGUbGBcqGHRaWkcqaIXaqmaaGccqGGSaalaeaacqWGybawcqGH9aqpcqWG4baEcqGHflY1cqWG4baEdaahaaWcbeqaaiabdsfaubaakiabg2da9maadmaabaqbaeqabiGaaaqaaiabigdaXaqaaiabdMha5naaCaaaleqabaGaemivaqfaaaGcbaGaemyEaKhabaGaemyEaKNaemyEaK3aaWbaaSqabeaacqWGubavaaaaaaGccaGLBbGaayzxaaGaeyicI4Sae8xhHi1aaWbaaSqabeaacqGGOaakcqWGUbGBcqGHRaWkcqaIXaqmcqGGPaqkcqGHxdaTcqGGOaakcqWGUbGBcqGHRaWkcqaIXaqmcqGGPaqkaaaaaOGaeiOla4IaaCzcaiaaxMaadaqadaqaaiabiwda1aGaayjkaiaawMcaaaaa@6D24@

Based on these definitions, the original problem (4), including both steady state equations and data consistency inequalities, can be rewritten as:

min⁡xxTQobjxs.t.xTQix=0L⋅x≥0x=[1y].     (6)
 MathType@MTEF@5@5@+=feaafiart1ev1aaatCvAUfKttLearuWrP9MDH5MBPbIqV92AaeXatLxBI9gBaebbnrfifHhDYfgasaacH8akY=wiFfYdH8Gipec8Eeeu0xXdbba9frFj0=OqFfea0dXdd9vqai=hGuQ8kuc9pgc9s8qqaq=dirpe0xb9q8qiLsFr0=vr0=vr0dc8meaabaqaciaacaGaaeqabaqabeGadaaakeaafaqadeabdaaaaeaadaWfqaqaaiGbc2gaTjabcMgaPjabc6gaUbWcbaGaemiEaGhabeaaaOqaaiabdIha4naaCaaaleqabaGaemivaqfaaOGaemyuae1aaSbaaSqaaiabd+gaVjabdkgaIjabdQgaQbqabaGccqWG4baEaeaaaeaacqqGZbWCcqGGUaGlcqqG0baDcqGGUaGlaeaacqWG4baEdaahaaWcbeqaaiabdsfaubaakiabdgfarnaaBaaaleaacqWGPbqAaeqaaOGaemiEaGhabaGaeyypa0JaeGimaadabaaabaGaemitaWKaeyyXICTaemiEaGhabaGaeyyzImRaeGimaadabaaabaGaemiEaGhabaGaeyypa0ZaamWaaeaafaqabeGabaaabaGaeGymaedabaGaemyEaKhaaaGaay5waiaaw2faaiabc6caUaaacaWLjaGaaCzcamaabmaabaGaeGOnaydacaGLOaGaayzkaaaaaa@5D4D@

Here, the symmetric matrix *Q*_*obj *_defines an objective function (e.g., the identity matrix), that selects a specific solution out of the possibly many equilibria. The symmetric matrices *Q*_*i *_correspond to the set of ODEs at steady state (Eqn. (1)) and the matrix *L *to linear inequality constraints derived, for instance, from approximate mass-balance equations (Eqn. (3)). Note, that in general the matrices *Q*_*i *_are a function of the set of (generally unknown) rate parameters *k*_*j*_, while *L*, which represents the mass balances, is not. The basic convex relaxation described in the *Appendix *then yields the following SDP relaxation of (6):

min⁡XTr(Qobj⋅X)s.t.Tr(Qi⋅X)=0Tr(e1⋅e1T⋅X)=1L⋅X⋅e1≥0L⋅X⋅LT≥0X≽0,     (7)
 MathType@MTEF@5@5@+=feaafiart1ev1aaatCvAUfKttLearuWrP9MDH5MBPbIqV92AaeXatLxBI9gBaebbnrfifHhDYfgasaacH8akY=wiFfYdH8Gipec8Eeeu0xXdbba9frFj0=OqFfea0dXdd9vqai=hGuQ8kuc9pgc9s8qqaq=dirpe0xb9q8qiLsFr0=vr0=vr0dc8meaabaqaciaacaGaaeqabaqabeGadaaakeaafaqadeGbdaaaaeaadaWfqaqaaiGbc2gaTjabcMgaPjabc6gaUbWcbaGaemiwaGfabeaaaOqaamXvP5wqSXMqHnxAJn0BKvguHDwzZbqegqvATv2CG4uz3bIuV1wyUbaceaGaa8hvaiaa=jhacqGGOaakcqWGrbqudaWgaaWcbaGaem4Ba8MaemOyaiMaemOAaOgabeaakiabgwSixlabdIfayjabcMcaPaqaaaqaaiabbohaZjabc6caUiabbsha0jabc6caUaqaaiaa=rfacaWFYbGaeiikaGIaemyuae1aaSbaaSqaaiabdMgaPbqabaGccqGHflY1cqWGybawcqGGPaqkaeaacqGH9aqpcqaIWaamaeaaaeaacaWFubGaa8NCaiabcIcaOiabdwgaLnaaBaaaleaacqaIXaqmaeqaaOGaeyyXICTaemyzau2aa0baaSqaaiabigdaXaqaaiabdsfaubaakiabgwSixlabdIfayjabcMcaPaqaaiabg2da9iabigdaXaqaaaqaaiabdYeamjabgwSixlabdIfayjabgwSixlabdwgaLnaaBaaaleaacqaIXaqmaeqaaaGcbaGaeyyzImRaeGimaadabaaabaGaemitaWKaeyyXICTaemiwaGLaeyyXICTaemitaW0aaWbaaSqabeaacqWGubavaaaakeaacqGHLjYScqaIWaamaeaaaeaacqWGybawaeaacqWI9=VBruWrPXgBtfMBZbacfaGae4hiaaIaeGimaaJaeiilaWcaaiaaxMaacaWLjaWaaeWaaeaacqaI3aWnaiaawIcacaGLPaaaaaa@93D8@

where Tr is the trace operator, which adds up the diagonal elements, and the inequality in the last line indicates that the matrix *X *must be positive semidefinite, i.e., all its eigenvalues should be greater than or equal to zero. The vector *e*_1 _is an all-zero vector, except for its first entry, which equals one to enforce *X*_11 _= 1. The set of feasible solutions, i.e., the set of matrices *X *that satisfy the constraints, is always a convex set. Recall that the matrix *X *as defined in Eqn. (5) is by construction a rank one matrix. This rank condition, however, is not guaranteed for the *X *obtained from the optimization, and thus this property has to be checked independently after a solution to the SDP is computed. This is necessary because the relaxed problem formulation (7) is less strict then the original form (6), hence the set of feasible solutions becomes larger. Note, that introduction of additional nonlinear constraints helps to reduce the set of " false positive" solutions, an aspect discussed in more detail in an additional section below. Finally, in the particular case of *Q*_*obj *_= 0, the problem reduces to whether or not the inequality can be satisfied for some matrix *X*. In this case, the SDP is referred to as a *feasibility problem*, where we are interested in proving the mere existence of solutions rather than finding any particular one.

### Dual SDP problems

The convexity of SDP has made it possible to develop sophisticated and reliable analytical and numerical methods to solve them [20]. A very important feature of SDP problems, from both the theoretical and applied viewpoints, is the associated *duality theory *(see also *Appendix*). For every SDP of the form (7) (usually called the *primal problem*), there is another associated SDP, called the *dual problem*, which can be derived via Lagrangian duality:

max⁡γs.t.Qobj≽γ⋅e1⋅e1T+∑iλi⋅Qi+e1⋅rT⋅L+LT⋅r⋅e1T+LT⋅S⋅L,     (8)
 MathType@MTEF@5@5@+=feaafiart1ev1aaatCvAUfKttLearuWrP9MDH5MBPbIqV92AaeXatLxBI9gBaebbnrfifHhDYfgasaacH8akY=wiFfYdH8Gipec8Eeeu0xXdbba9frFj0=OqFfea0dXdd9vqai=hGuQ8kuc9pgc9s8qqaq=dirpe0xb9q8qiLsFr0=vr0=vr0dc8meaabaqaciaacaGaaeqabaqabeGadaaakeaafaqaaeGacaaabaGagiyBa0MaeiyyaeMaeiiEaGhabaacciGae83SdCgabaGaee4CamNaeiOla4IaeeiDaqNaeiOla4cabaGaemyuae1aaSbaaSqaaiabd+gaVjabdkgaIjabdQgaQbqabaGccqWI9=VBcqWFZoWzcqGHflY1cqWGLbqzdaWgaaWcbaGaeGymaedabeaakiabgwSixlabdwgaLnaaDaaaleaacqaIXaqmaeaacqWGubavaaGccqGHRaWkdaaeqbqaaiab=T7aSnaaBaaaleaacqWGPbqAaeqaaOGaeyyXICTaemyuae1aaSbaaSqaaiabdMgaPbqabaGccqGHRaWkcqWGLbqzdaWgaaWcbaGaeGymaedabeaakiabgwSixlabdkhaYnaaCaaaleqabaGaemivaqfaaOGaeyyXICTaemitaWKaey4kaSIaemitaW0aaWbaaSqabeaacqWGubavaaGccqGHflY1cqWGYbGCcqGHflY1cqWGLbqzdaqhaaWcbaGaeGymaedabaGaemivaqfaaOGaey4kaSIaemitaW0aaWbaaSqabeaacqWGubavaaGccqGHflY1cqWGtbWucqGHflY1cqWGmbatcqGGSaalaSqaaiabdMgaPbqab0GaeyyeIuoaaaGccaWLjaGaaCzcamaabmaabaGaeGioaGdacaGLOaGaayzkaaaaaa@80A0@

where the constraint represents a matrix inequality with *r *≥ 0, *S *≥ 0, *S*_*ii *_= 0. The dual variables *γ*, *λ*, *r*, *S *are Lagrange multipliers associated to the different constraints in the primal problem. In the case of feasibility problems (i.e., *Q*_*obj *_= 0), the dual problem can be used to certify the nonexistence of solutions of the primal. This property will be crucial in our developments.

## Results

### Parameter estimation for nonlinear mass action kinetics at steady state

Identifying a satisfactory equilibrium point of a set of ODEs requires the solution of a system of algebraic equations (those that define the steady-state conditions) subject to additional equality or inequality constraints (consistency with experimental data). This can be time-demanding, because the system must often be simulated from a given set of initial conditions until it settles to an equilibrium [11,26]. This is particularly troublesome when the computed solution violates the experimental constraints and must hence be discarded. Traditional heuristic tools for this optimization problem require, for instance, an iterative procedure where the candidate sets of parameters are generated by some kind of wrapping parametrization algorithm (e.g., gradient-based or evolutionary), and a subsequent consistency check by integrating the system equations for these parameter values [19]. This trial and error method can be very time consuming because it only allows to check consistency in a subsequent step. Hence, an algorithm that searches for steady state solutions but is guaranteed to be consistent with all experimental data would be extremely valuable.

### SDP and nonlinear systems of equations

As opposed to these "indirect" techniques, our method is a conceptually novel direct approach based on a *convex relaxation *of the generalized parameter estimation problem at steady state (4). Our techniques apply whenever the model presentation is in polynomial form, since in this case the resulting system can be *relaxed *into a semidefinite programming problem (6), which in turn can be solved using efficient interior-point methods [27-30].

We illustrate the application of our approach with the following nonlinear system given by

[A]+[B]→k1[A·B][A·B]→k2[A·B∗][A·B∗]→k3[A]+[B].     (9)
 MathType@MTEF@5@5@+=feaafiart1ev1aaatCvAUfKttLearuWrP9MDH5MBPbIqV92AaeXatLxBI9gBaebbnrfifHhDYfgasaacH8akY=wiFfYdH8Gipec8Eeeu0xXdbba9frFj0=OqFfea0dXdd9vqai=hGuQ8kuc9pgc9s8qqaq=dirpe0xb9q8qiLsFr0=vr0=vr0dc8meaabaqaciaacaGaaeqabaqabeGadaaakeaafaqadeWadaaabaGaei4waSLaemyqaeKaeiyxa0Laey4kaSIaei4waSLaemOqaiKaeiyxa0fabaWaa4ajaSqaaiabdUgaRnaaBaaameaacqaIXaqmaeqaaaWcbeGccaGLsgcaaeaacqGGBbWwcqWGbbqqcqWIpM+zcqWGcbGqcqGGDbqxaeaacqGGBbWwcqWGbbqqcqWIpM+zcqWGcbGqcqGGDbqxaeaadaGdKaWcbaGaem4AaS2aaSbaaWqaaiabikdaYaqabaaaleqakiaawkziaaqaaiabcUfaBjabdgeabjabl+y6NjabdkeacnaaCaaaleqabaGaey4fIOcaaOGaeiyxa0fabaGaei4waSLaemyqaeKaeS4JPFMaemOqai0aaWbaaSqabeaacqGHxiIkaaGccqGGDbqxaeaadaGdKaWcbaGaem4AaS2aaSbaaWqaaiabiodaZaqabaaaleqakiaawkziaaqaaiabcUfaBjabdgeabjabc2faDjabgUcaRiabcUfaBjabdkeacjabc2faDjabc6caUaaacaWLjaGaaCzcamaabmaabaGaeGyoaKdacaGLOaGaayzkaaaaaa@6C39@

Here, two components *A *and *B *form a complex, *A *• *B*, that transforms into a modified form, *A *• B*, and finally decays into its building blocks. This system yields a set of four ODEs that at steady state reduces to a polynomial system:

[A]:−k1⋅[A]⋅[B]+k3⋅[A·B∗]=0[B]:−k1⋅[A]⋅[B]+k3⋅[A·B∗]=0[A·B]:k1⋅[A]⋅[B]−k2⋅[A·B]=0[A·B∗]:k2⋅[A·B]−k3⋅[A·B∗]=0.     (10)
 MathType@MTEF@5@5@+=feaafiart1ev1aaatCvAUfKttLearuWrP9MDH5MBPbIqV92AaeXatLxBI9gBaebbnrfifHhDYfgasaacH8akY=wiFfYdH8Gipec8Eeeu0xXdbba9frFj0=OqFfea0dXdd9vqai=hGuQ8kuc9pgc9s8qqaq=dirpe0xb9q8qiLsFr0=vr0=vr0dc8meaabaqaciaacaGaaeqabaqabeGadaaakeaafaqadeabdaaaaeaacqGGBbWwcqWGbbqqcqGGDbqxcqGG6aGoaeaacqGHsislcqWGRbWAdaWgaaWcbaGaeGymaedabeaakiabgwSixlabcUfaBjabdgeabjabc2faDjabgwSixlabcUfaBjabdkeacjabc2faDjabgUcaRiabdUgaRnaaBaaaleaacqaIZaWmaeqaaOGaeyyXICTaei4waSLaemyqaeKaeS4JPFMaemOqai0aaWbaaSqabeaacqGHxiIkaaGccqGGDbqxaeaacqGH9aqpcqaIWaamaeaacqGGBbWwcqWGcbGqcqGGDbqxcqGG6aGoaeaacqGHsislcqWGRbWAdaWgaaWcbaGaeGymaedabeaakiabgwSixlabcUfaBjabdgeabjabc2faDjabgwSixlabcUfaBjabdkeacjabc2faDjabgUcaRiabdUgaRnaaBaaaleaacqaIZaWmaeqaaOGaeyyXICTaei4waSLaemyqaeKaeS4JPFMaemOqai0aaWbaaSqabeaacqGHxiIkaaGccqGGDbqxaeaacqGH9aqpcqaIWaamaeaacqGGBbWwcqWGbbqqcqWIpM+zcqWGcbGqcqGGDbqxcqGG6aGoaeaacqWGRbWAdaWgaaWcbaGaeGymaedabeaakiabgwSixlabcUfaBjabdgeabjabc2faDjabgwSixlabcUfaBjabdkeacjabc2faDjabgkHiTiabdUgaRnaaBaaaleaacqaIYaGmaeqaaOGaeyyXICTaei4waSLaemyqaeKaeS4JPFMaemOqaiKaeiyxa0fabaGaeyypa0JaeGimaadabaGaei4waSLaemyqaeKaeS4JPFMaemOqai0aaWbaaSqabeaacqGHxiIkaaGccqGGDbqxcqGG6aGoaeaacqWGRbWAdaWgaaWcbaGaeGOmaidabeaakiabgwSixlabcUfaBjabdgeabjabl+y6Njabdkeacjabc2faDjabgkHiTiabdUgaRnaaBaaaleaacqaIZaWmaeqaaOGaeyyXICTaei4waSLaemyqaeKaeS4JPFMaemOqai0aaWbaaSqabeaacqGHxiIkaaGccqGGDbqxaeaacqGH9aqpcqaIWaamcqGGUaGlaaGaaCzcaiaaxMaadaqadaqaaiabigdaXiabicdaWaGaayjkaiaawMcaaaaa@C326@

If it is furthermore known from experimental data that in equimolar concentrations of *A *and *B *one third of them is unbound while the rest is associated in one of both complexes, we can write mass balances as

[A]=13,[B]=13,[A·B]+[A·B∗]=23,     (11)
 MathType@MTEF@5@5@+=feaafiart1ev1aaatCvAUfKttLearuWrP9MDH5MBPbIqV92AaeXatLxBI9gBaebbnrfifHhDYfgasaacH8akY=wiFfYdH8Gipec8Eeeu0xXdbba9frFj0=OqFfea0dXdd9vqai=hGuQ8kuc9pgc9s8qqaq=dirpe0xb9q8qiLsFr0=vr0=vr0dc8meaabaqaciaacaGaaeqabaqabeGadaaakeaafaqabeqadaaabaGaei4waSLaemyqaeKaeiyxa0Laeyypa0ZaaSaaaeaacqaIXaqmaeaacqaIZaWmaaGaeiilaWcabaGaei4waSLaemOqaiKaeiyxa0Laeyypa0ZaaSaaaeaacqaIXaqmaeaacqaIZaWmaaGaeiilaWcabaGaei4waSLaemyqaeKaeS4JPFMaemOqaiKaeiyxa0Laey4kaSIaei4waSLaemyqaeKaeS4JPFMaemOqai0aaWbaaSqabeaacqGHxiIkaaGccqGGDbqxcqGH9aqpdaWcaaqaaiabikdaYaqaaiabiodaZaaacqGGSaalaaGaaCzcaiaaxMaadaqadaqaaiabigdaXiabigdaXaGaayjkaiaawMcaaaaa@5428@

which have to be kept within a certain experimental accuracy *ε *= 20%.

The example above is thus a system of nonlinear polynomial relations based on mass action kinetics that comprises both equalities (Eqns. (10)) and inequalities (Eqns. (11)). As explained earlier, we define the state variables

*y*^*T *^= ([*A*], [*B*], [*A *• *B*], [*A *• B*]),     (12)

which are then used to produce a new matrix variable *X *according to Eqn. (5). To write the SDP problem, the equality and inequality constraints, Eqns. (10) and Eqns. (11), respectively, have to be rewritten into the form of Eqn. (6). For example, the steady state equation for species *B *becomes

QB=(00000.5⋅k300−0.5⋅k1000−0.5⋅k1000000000.5⋅k30000),     (13)
 MathType@MTEF@5@5@+=feaafiart1ev1aaatCvAUfKttLearuWrP9MDH5MBPbIqV92AaeXatLxBI9gBaebbnrfifHhDYfgasaacH8akY=wiFfYdH8Gipec8Eeeu0xXdbba9frFj0=OqFfea0dXdd9vqai=hGuQ8kuc9pgc9s8qqaq=dirpe0xb9q8qiLsFr0=vr0=vr0dc8meaabaqaciaacaGaaeqabaqabeGadaaakeaacqWGrbqudaWgaaWcbaacbiGae8Nqaieabeaakiabg2da9maabmaabaqbaeqabuqbaaaaaeaacqaIWaamaeaacqaIWaamaeaacqaIWaamaeaacqaIWaamaeaacqaIWaamcqGGUaGlcqaI1aqncqGHflY1cqWGRbWAdaWgaaWcbaGaeG4mamdabeaaaOqaaiabicdaWaqaaiabicdaWaqaaiabgkHiTiabicdaWiabc6caUiabiwda1iabgwSixlabdUgaRnaaBaaaleaacqaIXaqmaeqaaaGcbaGaeGimaadabaGaeGimaadabaGaeGimaadabaGaeyOeI0IaeGimaaJaeiOla4IaeGynauJaeyyXICTaem4AaS2aaSbaaSqaaiabigdaXaqabaaakeaacqaIWaamaeaacqaIWaamaeaacqaIWaamaeaacqaIWaamaeaacqaIWaamaeaacqaIWaamaeaacqaIWaamaeaacqaIWaamaeaacqaIWaamcqGGUaGlcqaI1aqncqGHflY1cqWGRbWAdaWgaaWcbaGaeG4mamdabeaaaOqaaiabicdaWaqaaiabicdaWaqaaiabicdaWaqaaiabicdaWaaaaiaawIcacaGLPaaacqGGSaalcaWLjaGaaCzcamaabmaabaGaeGymaeJaeG4mamdacaGLOaGaayzkaaaaaa@6B38@

while the rows of *L *corresponding to the mass balance inequalities for component *A *are

LA=(−1/3⋅(1−ε)10001/3⋅(1+ε)−1000).     (14)
 MathType@MTEF@5@5@+=feaafiart1ev1aaatCvAUfKttLearuWrP9MDH5MBPbIqV92AaeXatLxBI9gBaebbnrfifHhDYfgasaacH8akY=wiFfYdH8Gipec8Eeeu0xXdbba9frFj0=OqFfea0dXdd9vqai=hGuQ8kuc9pgc9s8qqaq=dirpe0xb9q8qiLsFr0=vr0=vr0dc8meaabaqaciaacaGaaeqabaqabeGadaaakeaacqWGmbatdaWgaaWcbaGaemyqaeeabeaakiabg2da9maabmaabaqbaeqabiqbaaaabaGaeyOeI0IaeGymaeJaei4la8IaeG4mamJaeyyXIC9aaeWaaeaacqaIXaqmcqGHsisliiGacqWF1oqzaiaawIcacaGLPaaaaeaacqaIXaqmaeaacqaIWaamaeaacqaIWaamaeaacqaIWaamaeaacqaIXaqmcqGGVaWlcqaIZaWmcqGHflY1daqadaqaaiabigdaXiabgUcaRiab=v7aLbGaayjkaiaawMcaaaqaaiabgkHiTiabigdaXaqaaiabicdaWaqaaiabicdaWaqaaiabicdaWaaaaiaawIcacaGLPaaacqGGUaGlcaWLjaGaaCzcamaabmaabaGaeGymaeJaeGinaqdacaGLOaGaayzkaaaaaa@54D4@

The feasible set of the relaxed SDP problem (7) is always convex and includes all the equilibria of the original problem. Thus, SDP optimization enables the possibility of directly solving the underlying nonlinear system of algebraic equations, or of proving its inconsistency. Furthermore, this property makes possible a direct consistency check based on feasibility or infeasibility of the SDP optimization. Typically, a parameterization approach would require identification of a stable steady state, either by root-finding or numerical integration, at which point the simulation can be verified or falsified for a given set of parameters *k*. In contrast, the SDP-based approach bypasses this time consuming step: if the problem (7) is infeasible the parameters *k *are inconsistent with the experimental data, and if a feasible solution of rank one can be found, the given parameters are valid.

For our running example, consider a set of parameters, *k*_1 _= 3, *k*_2 _= 1 and *k*_3 _= 1, respectively, for which the SDP is feasible. Let the solution be

X=1/9⋅(9333331111311113111131111),     (15)
 MathType@MTEF@5@5@+=feaafiart1ev1aaatCvAUfKttLearuWrP9MDH5MBPbIqV92AaeXatLxBI9gBaebbnrfifHhDYfgasaacH8akY=wiFfYdH8Gipec8Eeeu0xXdbba9frFj0=OqFfea0dXdd9vqai=hGuQ8kuc9pgc9s8qqaq=dirpe0xb9q8qiLsFr0=vr0=vr0dc8meaabaqaciaacaGaaeqabaqabeGadaaakeaacqWGybawcqGH9aqpcqaIXaqmcqGGVaWlcqaI5aqocqGHflY1daqadaqaauaabeqafuaaaaaabaGaeGyoaKdabaGaeG4mamdabaGaeG4mamdabaGaeG4mamdabaGaeG4mamdabaGaeG4mamdabaGaeGymaedabaGaeGymaedabaGaeGymaedabaGaeGymaedabaGaeG4mamdabaGaeGymaedabaGaeGymaedabaGaeGymaedabaGaeGymaedabaGaeG4mamdabaGaeGymaedabaGaeGymaedabaGaeGymaedabaGaeGymaedabaGaeG4mamdabaGaeGymaedabaGaeGymaedabaGaeGymaedabaGaeGymaedaaaGaayjkaiaawMcaaiabcYcaSiaaxMaacaWLjaWaaeWaaeaacqaIXaqmcqaI1aqnaiaawIcacaGLPaaaaaa@52F4@

which is rank one. Using the largest eigenvalue of *X *to scale the corresponding eigenvector yields

xsolT
 MathType@MTEF@5@5@+=feaafiart1ev1aaatCvAUfKttLearuWrP9MDH5MBPbIqV92AaeXatLxBI9gBaebbnrfifHhDYfgasaacH8akY=wiFfYdH8Gipec8Eeeu0xXdbba9frFj0=OqFfea0dXdd9vqai=hGuQ8kuc9pgc9s8qqaq=dirpe0xb9q8qiLsFr0=vr0=vr0dc8meaabaqaciaacaGaaeqabaqabeGadaaakeaacqWG4baEdaqhaaWcbaGaem4CamNaem4Ba8MaemiBaWgabaGaemivaqfaaaaa@33BA@ = (1,1/3,1/3,1/3,1/3) and ysolT
 MathType@MTEF@5@5@+=feaafiart1ev1aaatCvAUfKttLearuWrP9MDH5MBPbIqV92AaeXatLxBI9gBaebbnrfifHhDYfgasaacH8akY=wiFfYdH8Gipec8Eeeu0xXdbba9frFj0=OqFfea0dXdd9vqai=hGuQ8kuc9pgc9s8qqaq=dirpe0xb9q8qiLsFr0=vr0=vr0dc8meaabaqaciaacaGaaeqabaqabeGadaaakeaacqWG5bqEdaqhaaWcbaGaem4CamNaem4Ba8MaemiBaWgabaGaemivaqfaaaaa@33BC@ = (1/3,1/3,1/3,1/3).

A simple check shows that ysolT
 MathType@MTEF@5@5@+=feaafiart1ev1aaatCvAUfKttLearuWrP9MDH5MBPbIqV92AaeXatLxBI9gBaebbnrfifHhDYfgasaacH8akY=wiFfYdH8Gipec8Eeeu0xXdbba9frFj0=OqFfea0dXdd9vqai=hGuQ8kuc9pgc9s8qqaq=dirpe0xb9q8qiLsFr0=vr0=vr0dc8meaabaqaciaacaGaaeqabaqabeGadaaakeaacqWG5bqEdaqhaaWcbaGaem4CamNaem4Ba8MaemiBaWgabaGaemivaqfaaaaa@33BC@ directly fulfills the steady state condition and all additional constraints.

### Additional nonlinear constraints

The decision variable *X *in Eqn. (5) is by construction a rank one matrix. This property of *X*, however, is not necessarily guaranteed when solving the SDP relaxation (7) (including this constraint would destroy convexity). Hence, it has to be verified independently once the optimization is completed. From our numerical experience, a very limited number of solutions fails the rank one condition such that the corresponding results have to be discarded. A simple but effective combination of constraints, however, allows to reduce the number of these "false positive" solutions: since most of the inequality constraints are linear (e.g., mass balance equations), they can be used to tighten the description of the feasible set by using redundant constraints. By exhaustive multiplication of *m *pairs of linear inequality constraints, i.e. each upper bound is multiplied with every possible lower bound, (m2)
 MathType@MTEF@5@5@+=feaafiart1ev1aaatCvAUfKttLearuWrP9MDH5MBPbIqV92AaeXatLxBI9gBaebbnrfifHhDYfgasaacH8akY=wiFfYdH8Gipec8Eeeu0xXdbba9frFj0=OqFfea0dXdd9vqai=hGuQ8kuc9pgc9s8qqaq=dirpe0xb9q8qiLsFr0=vr0=vr0dc8meaabaqaciaacaGaaeqabaqabeGadaaakeaadaqadaqaauaabeqaceaaaeaacqWGTbqBaeaacqaIYaGmaaaacaGLOaGaayzkaaaaaa@3097@ new quadratic inequalities are generated. This multiplication of constraints corresponds to the term *L*·*X*·*L*^*T *^appearing in (7). Note, that even the product of a lower and an upper bound of the same constraint can be helpful, because the thus generated matrix has nonzero diagonal (i.e., quadratic) elements. These additional nonlinear algebraic constraints improve the performance of the algorithm significantly, because the set of feasible solutions that do not meet the rank one condition becomes much smaller.

### Analyzing whole regions of parameter space

The approach presented in the previous paragraphs can directly handle nonlinear algebraic equations in polynomial form. However, it considers only *single *sets of parameters, that have to be provided by an external parameter estimation algorithm. Since in general the parameter space can be quite large, it would be very helpful to be able to discard *a priori *large regions of the space, where we know for sure that no consistent rates can be found. As we will see, we can achieve this goal in a very efficient way by considering the dual optimization problem (8) presented earlier.

As in general convex optimization, infeasibility of the primal problem can be proven by the existence of a dual feasible solution, and conversely, dual infeasibility follows from a primal solution. Thus, the existence of dual variables satisfying the dual constraints in (8) directly proves primal infeasibility. This criterion can be used to yield an efficient procedure for the analysis of the parameter space. Therein, large regions can be detected where the given model is inconsistent with the experimental data. The feasible region will usually represent a small fraction in the overall parameter space. Therefore an efficient search of the parameter space will focus rather on negative (inconsistent) than positive (consistent) regions.

The use of SDP-based parametrizations allows to obtain exactly this information. Once dual feasibility has been proven for a given set of parameters, this point in the solution space is associated to specific values of the dual variables. This information can then be used to extend the feasibility check to larger regions in the parameter space. Recall that only the matrices *Q*_*i*_, i.e., the mass action kinetics, are actually dependent on the rate parameters *k*. We thus used a *bisection *approach in which the current parameter set is an *n*-dimensional box. Since this is a polytope, it follows that if the conditions are valid on the corners then they are also valid on the interior, and thus can be neglected for further analysis. In particular, we determine a factor *η *by which each parameter can be perturbed such that dual feasibility holds. We remark that the choice of cuboid-like regions is only a matter of convenience, the results can be easily extended in a direct fashion to any other polyhedral partition scheme (for instance, using simplices).

A formal proof of the guaranteed inconsistency of whole regions is provided in the *Appendix*. The pseudo-code of the complete algorithm for an efficient classification of the parameter space in feasible and infeasible regions is shown in Table 1. The procedure extends the infeasibility information from an isolated set of parameters to larger regions of parameter space by using SDP optimization. All parameter sets which lie in these extended regions need not to be considered any longer for feasibility purposes. Even more, in contrast to common grid-like discretization approaches, the algorithm allows an efficient and continuous exploration of the parameter space without undergoing the danger of missing a feasible solution by accident.

**Table 1:** Pseudo-code for efficient classification of the parameter space.

let *K *⊂ ℝ^*n *^be the parameter space
begin: set K* = *K *and *K*_*inconsistent*_* = ∅*
while (K* ≠ ∅)
consider loop *i*
generate parameter subset (*k*_*i *_± Δ*k*) ∈ K*
perform dual SDP optimization
if solution (*γ*, *λ*_*i*_, *P*, *r*, *S*) exists
// *k*_*i *_is dual feasible/primal infeasible
while (dual feasibility holds)
increase Δ*k*_*i*_
end
K* = K*\(*k*_*i *_± Δ*k*_*i*_)
*K*_*inconsistent *_= *K*_*inconsistent *_∪ (*k*_*i *_± Δ*k*_*i*_)
end
end

Algorithm for exploration of the parameter space. The set *K*_*inconsistent *_contains parameter values for which the equations and the measured date are incompatible.

### Efficient classification of the parameter space

**Figure 1:**
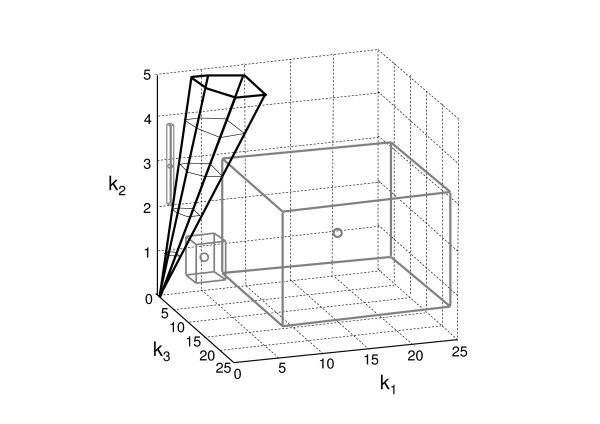
**Graph of the feasible parameter space**. Contour plot of the three-dimensional parameter space of *k*_1_, *k*_2 _and *k*_3 _from the example given in the text. The cone with the black edges marks the feasible region, infeasible areas are illustrated as cuboids with gray edges. Gray circles represent the original set of parameters where the bisection search started.

**Figure 2:**
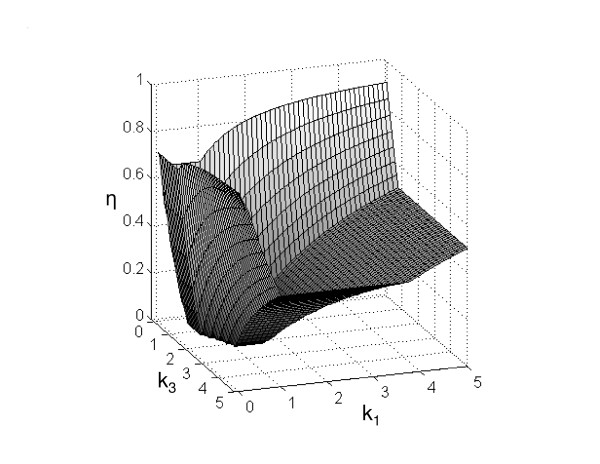
**Maximal possible parameter perturbation**. The size *η *of the maximal possible perturbation in the example given in the text is shown as a function of *k*_1 _and *k*_3 _(here, *k*_2 _= 1). The size of the perturbation increases with the distance from the feasible region and can be used to further refine the search directions in parameter space.

The results in Fig. 1 correspond to the example discussed earlier (further examples are shown in the *Appendix*). In the figure, the contour plot representing the feasible and infeasible regions in the 3-dimensional parameter space was obtained by exhaustive evaluation of sets of parameters. Particular solutions obtained with the SDP-based algorithm that prove infeasibility of whole regions are indicated in the graph. The respective regions of feasibility and infeasibility are shown, as well as a few cuboids that illustrate how we can discard the existence of solutions on whole regions of parameter space. Each box is obtained from the solution of a single small SDP optimization problem. It is clear that the SDP-based search in the infeasible region of the parameter space provides a powerful analysis tool, as it allows the efficient exploration of whole areas instead of the time consuming consistency check at single points. The figure also shows how the size of the boxes varies depending on the relative position (Fig. 1). In other words, the allowed perturbation *η *increases with the distance from the feasible region. This is highlighted in Fig. 2, where the distribution of *η *for one slice of the feasible region (in the *k*_1 _- *k*_3 _plane) is shown. Finally, since the systems of equations and inequalities are all linear in *k*, the feasible region is invariant under non-negative scalings (i.e., it is a cone), as can be seen in the three-dimensional plot in Fig. 1.

### Rigorous proofs for model discrimination

**Figure 3:**
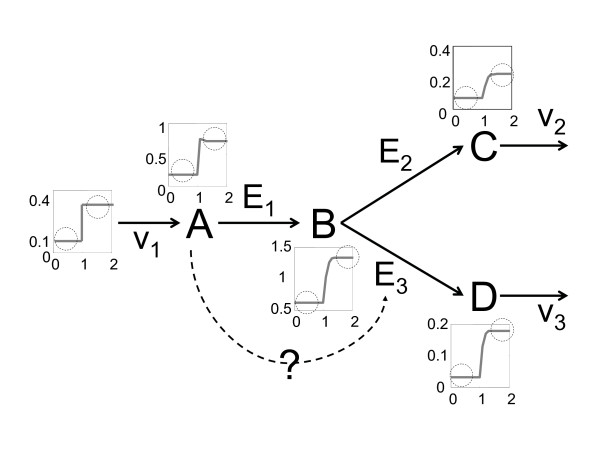
**Uncertain model structure**. It is unknown whether *A *exerts a positive influence on the conversion of *B *to *D*. By numerical integration of model alternative II, concentrations were obtained and used in lieu of experimental data. Overall component concentrations, a step response upon an increase of *v*_1 _and corresponding steady states (circle) are indicated in the inlays next to each component.

The basic algorithm for exploration of the parameter space can also be used to discriminate between different model alternatives, for instance when the system structure is uncertain [14]. This is possible since SDP allows for the exploration of the complete solution space. If this is the case, the failure of parametrization (i.e., the lack of suitable values for the model parameters) directly proves model inconsistency. Moreover, we avoid the danger of missing possible solutions by accident since we consider whole regions of parameter space instead of isolated points. As an example, consider a branching point in a metabolic pathway (Fig. 3). It is known that all conversion steps are catalyzed by enzymes (*E*_*i*_). However, while the reaction scheme for the pathway from *A *to *C *via *B *is well established, it may not be known whether the conversion from *B *to *D *is catalyzed by enzyme *E*_3 _or whether component *A *exerts some cooperative effect (Fig. 3). To discriminate between these two scenarios, two model alternatives that describe possible reaction schemes can be proposed (Table 2).

**Table 2:** Comparison of model alternatives for enzymatic conversion of *B *to *D*.

Model alternative I:
[B]+[E3]⇄koff,3kon,3[B·E3]→kcat,3[D]+[E3] MathType@MTEF@5@5@+=feaafiart1ev1aaatCvAUfKttLearuWrP9MDH5MBPbIqV92AaeXatLxBI9gBaebbnrfifHhDYfgasaacH8akY=wiFfYdH8Gipec8Eeeu0xXdbba9frFj0=OqFfea0dXdd9vqai=hGuQ8kuc9pgc9s8qqaq=dirpe0xb9q8qiLsFr0=vr0=vr0dc8meaabaqaciaacaGaaeqabaqabeGadaaakeaafaqaaeqafaaaaeaacqGGBbWwcqWGcbGqcqGGDbqxcqGHRaWkcqGGBbWwcqWGfbqrdaWgaaWcbaGaeG4mamdabeaakiabc2faDbqaamaao0aaleaacqWGRbWAdaWgaaadbaGaem4Ba8MaemOBa4MaeiilaWIaeG4mamdabeaaaSqaaiabdUgaRnaaBaaameaacqWGVbWBcqWGMbGzcqWGMbGzcqGGSaalcqaIZaWmaeqaaaGccaGLsgIaayjKHaaabaGaei4waSLaemOqaiKaeS4JPFMaemyrau0aaSbaaSqaaiabiodaZaqabaGccqGGDbqxaeaadaGdKaWcbaGaem4AaS2aaSbaaWqaaiabdogaJjabdggaHjabdsha0jabcYcaSiabiodaZaqabaaaleqakiaawkziaaqaaiabcUfaBjabdseaejabc2faDjabgUcaRiabcUfaBjabdweafnaaBaaaleaacqaIZaWmaeqaaOGaeiyxa0faaaaa@60D3@
Model alternative II:
[B]+[E3]⇄koff,3kon,3[B·E3] MathType@MTEF@5@5@+=feaafiart1ev1aaatCvAUfKttLearuWrP9MDH5MBPbIqV92AaeXatLxBI9gBaebbnrfifHhDYfgasaacH8akY=wiFfYdH8Gipec8Eeeu0xXdbba9frFj0=OqFfea0dXdd9vqai=hGuQ8kuc9pgc9s8qqaq=dirpe0xb9q8qiLsFr0=vr0=vr0dc8meaabaqaciaacaGaaeqabaqabeGadaaakeaafaqaaeqadaaabaGaei4waSLaemOqaiKaeiyxa0Laey4kaSIaei4waSLaemyrau0aaSbaaSqaaiabiodaZaqabaGccqGGDbqxaeaadaGdnaWcbaGaem4AaS2aaSbaaWqaaiabd+gaVjabd6gaUjabcYcaSiabiodaZaqabaaaleaacqWGRbWAdaWgaaadbaGaem4Ba8MaemOzayMaemOzayMaeiilaWIaeG4mamdabeaaaOGaayPKHiaawcziaaqaaiabcUfaBjabdkeacjabl+y6NjabdweafnaaBaaaleaacqaIZaWmaeqaaOGaeiyxa0faaaaa@4E91@
[B·E3]+[A]⇄koff,3bkon,3b[B·E3·A]→kcat,3b[D]+[B·E3] MathType@MTEF@5@5@+=feaafiart1ev1aaatCvAUfKttLearuWrP9MDH5MBPbIqV92AaeXatLxBI9gBaebbnrfifHhDYfgasaacH8akY=wiFfYdH8Gipec8Eeeu0xXdbba9frFj0=OqFfea0dXdd9vqai=hGuQ8kuc9pgc9s8qqaq=dirpe0xb9q8qiLsFr0=vr0=vr0dc8meaabaqaciaacaGaaeqabaqabeGadaaakeaafaqaaeqafaaaaeaacqGGBbWwcqWGcbGqcqWIpM+zcqWGfbqrdaWgaaWcbaGaeG4mamdabeaakiabc2faDjabgUcaRiabcUfaBjabdgeabjabc2faDbqaamaao0aaleaacqWGRbWAdaWgaaadbaGaem4Ba8MaemOBa4MaeiilaWIaeG4mamJaemOyaigabeaaaSqaaiabdUgaRnaaBaaameaacqWGVbWBcqWGMbGzcqWGMbGzcqGGSaalcqaIZaWmcqWGIbGyaeqaaaGccaGLsgIaayjKHaaabaGaei4waSLaemOqaiKaeS4JPFMaemyrau0aaSbaaSqaaiabiodaZaqabaGccqWIpM+zcqWGbbqqcqGGDbqxaeaadaGdKaWcbaGaem4AaS2aaSbaaWqaaiabdogaJjabdggaHjabdsha0jabcYcaSiabiodaZiabdkgaIbqabaaaleqakiaawkziaaqaaiabcUfaBjabdseaejabc2faDjabgUcaRiabcUfaBjabdkeacjabl+y6NjabdweafnaaBaaaleaacqaIZaWmaeqaaOGaeiyxa0faaaaa@6F2D@

**Figure 4:**
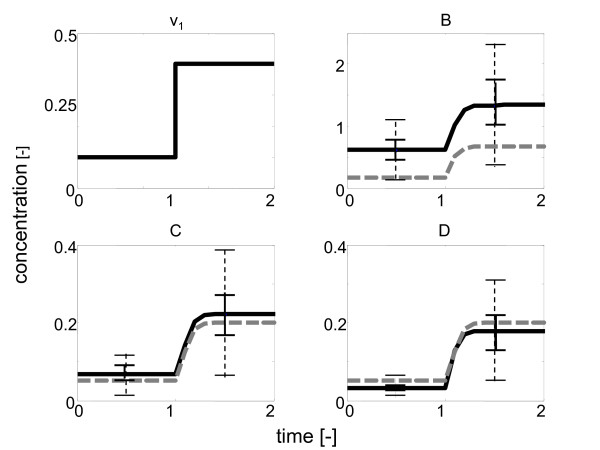
**Model discrimination based on two distinct steady states**. The figure shows variations in *v*_1 _simulated with model I (gray dashed line) and model II (black solid line), respectively. A large standard deviation of the experimental data (*ε *= 0.75, dashed error bars) has to be allowed for model I, otherwise mass balances are violated (*ε *= 0.25, solid error bars). For the simulations, the constants for complex formation were set to the following values: association *k*_on,*i *_= 1; dissociation *k*_off _= 0.1; catalytic step *k*_cat,*i *_= 1. During the parametrization step, only the association constants of the complexes were estimated in a range from 0.3 to 3.

The consistency of both models is analyzed through defined variations in the incoming carbon flux *v*_*i*_: An artificial data set is generated by simulation of model II, that in turn is used for parameter analysis (Figs. 3 and 4). Hence, two steady states can be simultaneously considered for SDP optimization. As expected, parameter estimation of model II identifies the feasible region in the solution space without any problems. In contrast, no feasible set of parameters is found for model I when an experimental error of *ε *= 0.25 is assumed (Fig. 4). This is a direct proof that model I is incorrect and needs to be modified. Only when an intolerably high deviation of *ε *= 0.75 is allowed, the parametrization algorithm finds solutions that are consistent with the experimental data (Fig. 4). The basic ideas underlying this small example can be directly applied to real biological problem sets where the structure is unknown. Since the search of the parameter space can be completed in a few steps, SDP provides a powerful tool for model discrimination and can be used for model invalidation and subsequent redesign of experimental set-ups.

## Discussion

We introduced a conceptually novel approach for parameter estimation and parameter space classification based on a direct search for solutions that are simultaneously consistent with the steady-state concentrations and the inequalities arising from experimental data. The method is based on semidefinite optimization, whose convexity properties allow for the direct solution (of relaxations) of nonlinear optimization problems in polynomial form. It is thus possible to establish the possible infeasibility of steady state concentrations for a given set of parameters under direct consideration of experimental constraints. Our approach avoids the time-consuming numerical identification of a stable steady state, where feasibility can only be validated in retrospective [11,26]. Besides the direct consistency analysis for single sets of parameters, the duality properties of SDP optimization problems allow the direct proof of feasibility (or infeasibility) of whole regions in the parameter space instead of the mere consideration of isolated spots. This significantly reduces the total number of possibilities that have to be evaluated. Moreover, our approach is based on a simple convex relaxation of the original parametrization problem and can hence easily be applied without time consuming problem reformulation.

The possibility of discarding whole regions of the parameter space from further exploration is extremely valuable for model discrimination, where determination of model consistency or inconsistency with experimental data is the desired goal [14]. This approach can be very time-intensive due to the trial and error method that includes several integrations per set of parameters. Our algorithm thus provides an valuable tool for model discrimination.

Despite the immediate practical applications of our method, further work is necessary to fully exploit the total possibilities of the algorithm presented. Since the problem size increases with the number of variables of the system, the corresponding optimization problems will inevitably result in large matrices. Current versions of SDP solvers [27-30], however, slow down considerably when larger problem sets with more than about 30 state variables were analyzed. Stiffness of the underlying system of equations is another difficulty, which remains unsolved to date. Hence, problems which large differences in the kinetic parameters, e.g., if Michaelis-Menten kinetics are transformed to mass action kinetics, can probably not be solved without sophisticated preprocessing. An adequate way of scaling therefore needs to be developed. Additionally, the efficiency of the wrapping parametrization algorithm itself could also be improved, since currently there is no control level algorithm that supervises the search direction in the parameter space. Valuable information, however, is available, since the shape of the boxes indicates the maximal possible perturbation *η*, and this could be used to determine the direction of the next step (Fig. 2). A promising approach would thus be a method that simultaneously uses primal and dual information.

## Conclusion

In conclusion, we believe the results shown here are an important first step towards the integration of SDP as a tool to solve and analyze polynomial systems in chemical and biochemical engineering. Since our SDP-based algorithm allows to increase the efficiency of the pivotal steps in parameter estimation, it has great potential for the identification of nonlinear systems that prevail in biology.

## Competing interests

The authors declare that they have no competing interests.

## Authors' contributions

LK and PAP designed the study. LK conceived the modeling background. PAP derived and supervised the SDP part of the work. US participated in the design and coordinated the project. All authors read and approved the final manuscript.

## Appendix

### Convex relaxation

Consider a set of quadratic equations and linear inequalities for the vector *x *as defined in (5).

*x*^*T *^·*Q*_*i*_·*x *= 0,     *L*·*x *≥ *0*.     (16)

Defining *X *= *x*·*x*^*T*^, we find that these equations can be rewritten as *affine *expressions in the matrix *X*. A semidefinite relaxation of Eqns. (16) is then obtained by replacing the nonconvex constraint *X *= *x*·*x*^*T *^with the weaker (but convex) alternative:

*X*_11 _= 1,     *X *≥ 0,

yielding the system

Tr(*Q*_*i*_·*X*) = 0, *L*·*X*·*e*_1 _≥ 0,     (17)

with *X*_11 _= 1,     *X *≽ 0.

In general, the original (Eqns. (16)) and the new (Eqns. (17)) formulations are equivalent only if the matrix *X *has rank one. However, since such rank constraint is nonconvex, it cannot be directly included in the SDP optimization. This relaxation causes the set of solutions to becomes larger, and as a consequence the rank condition must be checked independently after the optimization is completed. Notice also that the formulation can be strengthened by adding the redundant constraints *L*·*X*·*L*^*T *^≥ 0.

### Weak Duality in SDP problems

A typical SDP problem in primal form is

min     Tr(*C*·*X*)

s.t     Tr(*A*_*i*_·*X*) = *b*_*i*_, *i *= 1, ..., *m *    (18)

*X *≽ 0.

The associated dual problem can be stated as

max⁡bTzs.t∑i=1mAizi⪯C,     (19)
 MathType@MTEF@5@5@+=feaafiart1ev1aaatCvAUfKttLearuWrP9MDH5MBPbIqV92AaeXatLxBI9gBaebbnrfifHhDYfgasaacH8akY=wiFfYdH8Gipec8Eeeu0xXdbba9frFj0=OqFfea0dXdd9vqai=hGuQ8kuc9pgc9s8qqaq=dirpe0xb9q8qiLsFr0=vr0=vr0dc8meaabaqaciaacaGaaeqabaqabeGadaaakeaafaqabeGacaaabaGagiyBa0MaeiyyaeMaeiiEaGhabaGaemOyai2aaWbaaSqabeaacqWGubavaaacbeGccqWF6bGEaeaaieaacqGFZbWCcqGFUaGlcqGF0baDaeaadaaeWaqaaiabdgeabnaaBaaaleaacqWGPbqAaeqaaaqaaiabdMgaPjabg2da9iabigdaXaqaaiabd2gaTbqdcqGHris5aOGaemOEaO3aaSbaaSqaaiabdMgaPbqabaGccqWI9=VBcqWGdbWqcqGGSaalaaGaaCzcaiaaxMaadaqadaqaaiabigdaXiabiMda5aGaayjkaiaawMcaaaaa@4ED5@

where *b *= (*b*_1_,...,*b*_*m*_), and the vector **z **= (*z*_1_,...,*z*_*m*_) contains the dual decision variables.

The key relationship between the primal and the dual problem is the fact that feasible solutions of one can be used to bound the values of the other problem. Indeed, let *X *and **z **be any two feasible solutions of the primal and dual problems respectively. Then we have the following inequality:

Tr(*C*·*X*) ≥ *b*^*T *^**z**.     (20)

This property is known as *weak duality*. Thus, we can use any feasible *X *to compute an upper bound for the optimum of *b*^*T *^**z**, and we can also use any feasible **z **to compute a lower bound for the optimum of Tr(*C*·*X*).

### Proof for regions of inconsistency

If a feasible dual solution can be found, Eqn. (8) will be satisfied. As noticed earlier, the matrices *Q*_*i *_explicitly depend on the unknown rates *k *in an affine way. Thus, we want to be able to disprove the consistency of not just one particular fixed value of the rate constants, but to *simultaneously *discard whole *sets *of rates at the same time. In other words, we want to find solutions (*γ*, *λ*_*i*_, *r*, *S*) of the dual form (8) that work for all rate constants *k *on a given set.

Notice that the dependence of the matrices *Q*_*i *_on the rate constants *k *is affine. Assume the nominal value and deviation of rate constant *j *is *k*_*j*0 _± Δ*k*_*j*_. Then, we can write

Qi(k)=Qi0+∑jQijδjΔkj,
 MathType@MTEF@5@5@+=feaafiart1ev1aaatCvAUfKttLearuWrP9MDH5MBPbIqV92AaeXatLxBI9gBaebbnrfifHhDYfgasaacH8akY=wiFfYdH8Gipec8Eeeu0xXdbba9frFj0=OqFfea0dXdd9vqai=hGuQ8kuc9pgc9s8qqaq=dirpe0xb9q8qiLsFr0=vr0=vr0dc8meaabaqaciaacaGaaeqabaqabeGadaaakeaacqWGrbqudaWgaaWcbaGaemyAaKgabeaakiabcIcaOiabdUgaRjabcMcaPiabg2da9iabdgfarnaaBaaaleaacqWGPbqAcqaIWaamaeqaaOGaey4kaSYaaabuaeaacqWGrbqudaWgaaWcbaGaemyAaKMaemOAaOgabeaaiiGakiab=r7aKnaaBaaaleaacqWGQbGAaeqaaOGaeuiLdqKaem4AaS2aaSbaaSqaaiabdQgaQbqabaGccqGGSaalaSqaaiabdQgaQbqab0GaeyyeIuoaaaa@481B@

where *δ*_*j *_:= (*k*_*j *_- *k*_*j*0_)/Δ*k*_*j*_, and |*δ*_*j*_| ≤ 1. To guarantee that the dual form (8) holds for all allowable values of the rate constants, we can use the following result:

**Lemma 1 ***Consider the linear matrix inequality given by:*

A0+∑i=1nδiAi≽0.     (21)
 MathType@MTEF@5@5@+=feaafiart1ev1aaatCvAUfKttLearuWrP9MDH5MBPbIqV92AaeXatLxBI9gBaebbnrfifHhDYfgasaacH8akY=wiFfYdH8Gipec8Eeeu0xXdbba9frFj0=OqFfea0dXdd9vqai=hGuQ8kuc9pgc9s8qqaq=dirpe0xb9q8qiLsFr0=vr0=vr0dc8meaabaqaciaacaGaaeqabaqabeGadaaakeaacqWGbbqqdaWgaaWcbaGaeGimaadabeaakiabgUcaRmaaqahabaacciGae8hTdq2aaSbaaSqaaiabdMgaPbqabaGccqWGbbqqdaWgaaWcbaGaemyAaKgabeaakiabl2==UjabicdaWiabc6caUiaaxMaacaWLjaWaaeWaaeaacqaIYaGmcqaIXaqmaiaawIcacaGLPaaaaSqaaiabdMgaPjabg2da9iabigdaXaqaaiabd6gaUbqdcqGHris5aaaa@460A@

If there exist η, W_i_, such that

A0=∑i=1nWi,{Wi+η⋅Ai≽0Wi−η⋅Ai≽0,     (22)
 MathType@MTEF@5@5@+=feaafiart1ev1aaatCvAUfKttLearuWrP9MDH5MBPbIqV92AaeXatLxBI9gBaebbnrfifHhDYfgasaacH8akY=wiFfYdH8Gipec8Eeeu0xXdbba9frFj0=OqFfea0dXdd9vqai=hGuQ8kuc9pgc9s8qqaq=dirpe0xb9q8qiLsFr0=vr0=vr0dc8meaabaqaciaacaGaaeqabaqabeGadaaakeaafaqabeqacaaabaGaemyqae0aaSbaaSqaaiabicdaWaqabaGccqGH9aqpdaaeWbqaaiabdEfaxnaaBaaaleaacqWGPbqAaeqaaOGaeiilaWcaleaacqWGPbqAcqGH9aqpcqaIXaqmaeaacqWGUbGBa0GaeyyeIuoaaOqaamaaceqabaqbaeqabiqaaaqaaiabdEfaxnaaBaaaleaacqWGPbqAaeqaaOGaey4kaSccciGae83TdGMaeyyXICTaemyqae0aaSbaaSqaaiabdMgaPbqabaGccqWI9=VBcqaIWaamaeaacqWGxbWvdaWgaaWcbaGaemyAaKgabeaakiabgkHiTiab=D7aOjabgwSixlabdgeabnaaBaaaleaacqWGPbqAaeqaaOGaeSy==7MaeGimaadaaaGaay5EaaGaeiilaWIaaCzcaiaaxMaadaqadaqaaiabikdaYiabikdaYaGaayjkaiaawMcaaaaaaaa@5DA8@

*for i *= 1,..., *n, then Eqn. (21) holds for all δ such that *|*δ*_*i*_| ≤ *η*.

The lemma follows easily from the identity:

A0+∑i=1nδiAi=12η∑i=1n(η+δi)(Wi+ηAi)+(η−δi)(Wi−ηAi).
 MathType@MTEF@5@5@+=feaafiart1ev1aaatCvAUfKttLearuWrP9MDH5MBPbIqV92AaeXatLxBI9gBaebbnrfifHhDYfgasaacH8akY=wiFfYdH8Gipec8Eeeu0xXdbba9frFj0=OqFfea0dXdd9vqai=hGuQ8kuc9pgc9s8qqaq=dirpe0xb9q8qiLsFr0=vr0=vr0dc8meaabaqaciaacaGaaeqabaqabeGadaaakeaacqWGbbqqdaWgaaWcbaGaeGimaadabeaakiabgUcaRmaaqahabaacciGae8hTdq2aaSbaaSqaaiabdMgaPbqabaGccqWGbbqqdaWgaaWcbaGaemyAaKgabeaakiabg2da9maalaaabaGaeGymaedabaGaeGOmaiJae83TdGgaamaaqahabaWaaeWaaeaacqWF3oaAcqGHRaWkcqWF0oazdaWgaaWcbaGaemyAaKgabeaaaOGaayjkaiaawMcaaaWcbaGaemyAaKMaeyypa0JaeGymaedabaGaemOBa4ganiabggHiLdGcdaqadaqaaiabdEfaxnaaBaaaleaacqWGPbqAaeqaaOGaey4kaSIae83TdGMaemyqae0aaSbaaSqaaiabdMgaPbqabaaakiaawIcacaGLPaaacqGHRaWkdaqadaqaaiab=D7aOjabgkHiTiab=r7aKnaaBaaaleaacqWGPbqAaeqaaaGccaGLOaGaayzkaaWaaeWaaeaacqWGxbWvdaWgaaWcbaGaemyAaKgabeaakiabgkHiTiab=D7aOjabdgeabnaaBaaaleaacqWGPbqAaeqaaaGccaGLOaGaayzkaaaaleaacqWGPbqAcqGH9aqpcqaIXaqmaeaacqWGUbGBa0GaeyyeIuoakiabc6caUaaa@6B87@

Since the expressions in (22) are affine in the unknowns *η*, *W*_*i*_, we can hence directly use this lemma to obtain guaranteed regions of inconsistency.

### Further case studies

Additional example 1:

Consider a simple two-element linear reaction, where component *A *is converted into component *B *and vice versa:

[A]⇄k2k1[B].
 MathType@MTEF@5@5@+=feaafiart1ev1aaatCvAUfKttLearuWrP9MDH5MBPbIqV92AaeXatLxBI9gBaebbnrfifHhDYfgasaacH8akY=wiFfYdH8Gipec8Eeeu0xXdbba9frFj0=OqFfea0dXdd9vqai=hGuQ8kuc9pgc9s8qqaq=dirpe0xb9q8qiLsFr0=vr0=vr0dc8meaabaqaciaacaGaaeqabaqabeGadaaakeaafaqabeqadaaabaGaei4waSLaemyqaeKaeiyxa0fabaWaa4qdaSqaaiabdUgaRnaaBaaameaacqaIXaqmaeqaaaWcbaGaem4AaS2aaSbaaWqaaiabikdaYaqabaaakiaawkzicaGLqgcaaeaacqGGBbWwcqWGcbGqcqGGDbqxcqGGUaGlaaaaaa@3C85@

**Figure 5:**
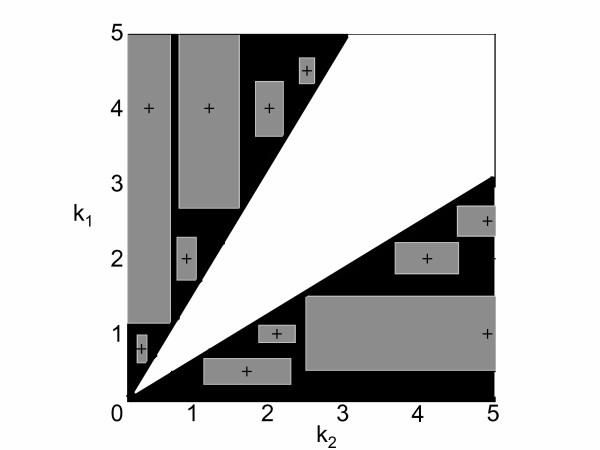
**Contour plot for additional example 1**. The feasible and infeasible regions are shown in white and black, respectively. Starting from an initial set of parameters (black cross), whole areas can be proven to be inconsistent (gray).

We assume unimolar concentrations of species *A *and *B *that are kept within an accuracy of 25%. The two-dimensional contour plot including feasible and infeasible regions is shown in Fig. 5.

Additional example 2:

Consider another simple nonlinear system, where *mRNA *binds to a ribosome to form a protein *P*, which decays at a certain rate:

[mRNA]+[rib]⇄k2k1[mRNA·rib][mRNA·rib]→k3[mRNA]+[rib]+[P][P]→k4∅.
 MathType@MTEF@5@5@+=feaafiart1ev1aaatCvAUfKttLearuWrP9MDH5MBPbIqV92AaeXatLxBI9gBaebbnrfifHhDYfgasaacH8akY=wiFfYdH8Gipec8Eeeu0xXdbba9frFj0=OqFfea0dXdd9vqai=hGuQ8kuc9pgc9s8qqaq=dirpe0xb9q8qiLsFr0=vr0=vr0dc8meaabaqaciaacaGaaeqabaqabeGadaaakeaafaqaaeWadaaabaGaei4waSLaemyBa0MaemOuaiLaemOta4KaemyqaeKaeiyxa0Laey4kaSIaei4waSLaemOCaiNaemyAaKMaemOyaiMaeiyxa0fabaWaa4qdaSqaaiabdUgaRnaaBaaameaacqaIXaqmaeqaaaWcbaGaem4AaS2aaSbaaWqaaiabikdaYaqabaaakiaawkzicaGLqgcaaeaacqGGBbWwcqWGTbqBcqWGsbGucqWGobGtcqWGbbqqcqWIpM+zcqWGYbGCcqWGPbqAcqWGIbGycqGGDbqxaeaacqGGBbWwcqWGTbqBcqWGsbGucqWGobGtcqWGbbqqcqWIpM+zcqWGYbGCcqWGPbqAcqWGIbGycqGGDbqxaeaadaGdKaWcbaGaem4AaS2aaSbaaWqaaiabiodaZaqabaaaleqakiaawkziaaqaaiabcUfaBjabd2gaTjabdkfasjabd6eaojabdgeabjabc2faDjabgUcaRiabcUfaBjabdkhaYjabdMgaPjabdkgaIjabc2faDjabgUcaRiabcUfaBjabdcfaqjabc2faDbqaaiabcUfaBjabdcfaqjabc2faDbqaamaaoqcaleaacqWGRbWAdaWgaaadbaGaeGinaqdabeaaaSqabOGaayPKHaaabaGaeyybIySaeiOla4caaaaa@8061@

**Figure 6:**
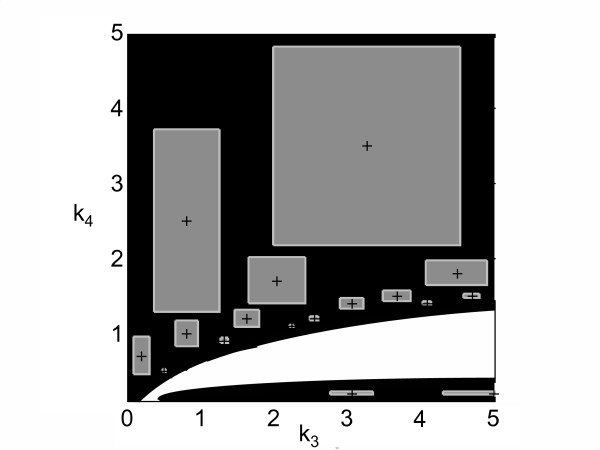
**Contour plot for additional example 2**. The feasible and infeasible regions are shown in white and black, respectively. Starting from an initial set of parameters (black cross), whole areas can be proven to be inconsistent (gray) (parameters *k*_1 _and *k*_2 _are fixed, *k*_3 _and *k*_4 _are varied in a range between 0 and 5).

For simplicity, the overall concentrations of *mRNA*, ribosome and *P *are all equal to 1 and have to be kept within an accuracy of *ε *= 25%. The overall parameter space is 4-dimensional. Fig. 6 shows the contour plot in the *k*_3 _- *k*_4 _plane.
